# Comparison between the Effects of Bretschneider’s HTK Solution and Cold Blood Cardioplegia on Systemic Endothelial Functions in Patients who Undergo Coronary Artery Bypass Surgery: a Prospective Randomized and Controlled Trial

**DOI:** 10.21470/1678-9741-2019-0327

**Published:** 2020

**Authors:** Ilker Mercan, Yuksel Dereli, Cemile Topcu, Omer Tanyeli, Mehmet Isik, Niyazi Gormus, Elifnur Yildirim Ozturk

**Affiliations:** 1 Department of Cardiovascular Surgery, Konya Training and Research Hospital, Konya, Turkey.; 2 Department of Cardiovascular Surgery, Necmettin Erbakan University, Meram Faculty of Medicine, Konya, Turkey.; 3 Department of Biochemistry, Necmettin Erbakan University, Meram Faculty of Medicine, Konya, Turkey.; 4 Department of Public Health, Necmettin Erbakan University, Meram Faculty of Medicine, Konya, Turkey.

**Keywords:** Bretschneider cardioplegic solution. Von Willebrand Factor. Histidine. Heart Arrest, Induced. Potassium Chloride. Coronary Artery Bypass

## Abstract

**Objective:**

To investigate the effects of Bretschneider’s histidine-tryptophan-ketoglutarate (HTK) solution and cold blood cardioplegia on systemic endothelial functions.

**Methods:**

A total of 50 patients who underwent isolated coronary artery bypass surgery between March 2018 and May 2018 were randomly divided into two groups - group 1 (Bretschneider’s HTK solution, n=25) and group 2 (cold blood cardioplegia, n=25). Data related to the indicators of endothelial dysfunction were recorded. Flow-mediated dilation was measured together with the assessment of the values of endothelin-1, von Willebrand factor, and asymmetric dimethylarginine to identify endothelial dysfunction. Then, the two groups were compared regarding these values.

**Results:**

The most significant result of our study was that the endothelin-1 level was significantly higher in group 2 than in group 1 (*P*<0.001). The value of flow-mediated dilation was found to increase to a lesser degree on the postoperative days compared to the value at the day of admission in group 1 (*P*=0.002 and *P*=0.030, respectively).

**Conclusion:**

Cardiopulmonary bypass leads to endothelial dysfunction. Our results revealed that Bretschneider’s HTK solution causes less severe endothelial injury than cold blood cardioplegia.

**Table:** 

Abbreviations, acronyms & symbols				
**ACE** **ACT** **ADMA** **AF** **ANOVA** **BMI** **CABG** **CBC** **COPD** **CPB** **DM** **EDHF** **ELISA** **ET-1**	**= Angiotensin-converting enzyme** **= Activated clotting time** **= Asymmetric dimethylarginine** **= Atrial fibrillation** **= Analysis of variance** **= Body mass index** **= Coronary artery bypass grafting** **= Cold blood cardioplegia** **= Chronic obstructive pulmonary disease** **= Cardiopulmonary bypass** **= Diabetes mellitus** **= Endothelium-derived hyperpolarizing factor** **= Enzyme-linked immunosorbent assay** **= Endothelin-1**	** **	**FMD** **HT** **HTK** **ICU** **IMA** **IU** **LAD** **LVEF** **NO** **PAD** **SD** **vWF** **WU**	**= Flow-mediated dilation** **= Arterial hypertension** **= Histidine-tryptophan-ketoglutarate** **= Intensive care unit** **= Internal mammary artery** **= International unit** **= Left anterior descending** **= Left ventricular ejection fraction** **= Nitric oxide** **= Peripheral arterial disease** **= Standard deviation** **= von Willebrand factor** **= Wisconsin University**

## INTRODUCTION

The development of cardiopulmonary bypass (CPB) systems is undoubtedly one of the most significant steps in cardiac surgery. However, numerous factors, mainly the inflammatory changes due to non-physiological blood flow within non-endothelialized lines, high-dose anticoagulation, together with gas and microparticle emboli, affect the end-organ perfusion adversely during CPB. The two most important causes of mortality following cardiac surgery are incompetent surgical technique and inadequate myocardial protection. Cardioplegia protocols, which are used for the provision of myocardial protection, have quite different variations regarding their components, routes, and doses of administration, duration of recurrent administration, and delivery temperatures^[1,2]^. Myocardial ischemia, developing after cardiac arrest due to cardioplegia, and ischemia-reperfusion injury due to initiation of physiological coronary perfusion following removal of the aortic clamp are among the most critical problems of CPB-guided cardiac surgery^[1,3]^.

Endothelial tissue covering the inner wall of the vessels regulates basic hemostatic functions such as vascular tone, circulation of blood cells, inflammation, and platelet activity by secreted proinflammatory and anti-inflammatory cytokines. All these events, which endothelium maintains in perfect balance, deteriorate when endothelial dysfunction develops, and many pathological conditions occur. It is obvious that CPB disrupts endothelial functions^[3]^.

While such controversy is going on, the effect of the type of cardioplegia on systemic endothelial functions is an important topic that deserves to be investigated. Due to its non-physiological circulatory pattern, cardiac operations performed under CPB lead to severe injury of the vascular endothelium^[4-6]^. Numerous methods have been developed for identification of endothelial dysfunction. The most commonly used bioindicators and tests with this purpose in the literature are endothelin-1 (ET-1), von Willebrand factor (vWF), asymmetric dimethylarginine (ADMA), and ultrasonographic assessment of flow-mediated dilation (FMD)^[7-10]^.

**Fig. 1:**
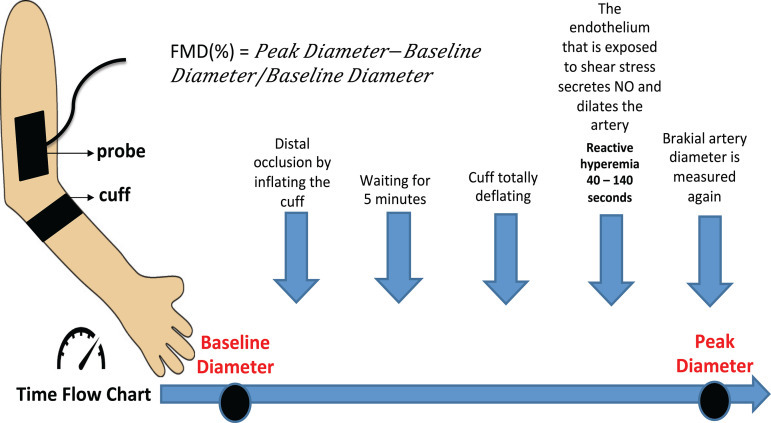
Flow-mediated dilation (FMD). NO=nitric oxide

Nitric oxide (NO) inhibits aggregation of platelets, adhesion of monocytes and leukocytes to the endothelium, in addition to inhibiting vascular inflammation by suppressing the adhesion molecules and chemokines. Decreased NO synthesis may cause endothelial dysfunction. ADMA is an inhibitor of NO synthase. It has been shown that ET-1 disrupts endothelium-dependent vasodilation and causes oxidative stress by increasing superoxide formation^[11]^. vWF is synthesized and stored by vascular endothelial cells and is involved in platelet aggregation and adhesion. Furthermore, NO may mediate a feedback inhibition in vWF secretion. Numerous studies have demonstrated the relationship between elevated vWF levels and endothelial dysfunction^[12]^. FMD is a less invasive technique. In this technique, brachial artery diameter is measured by ultrasound before and after an increase in shear stress that is induced by reactive hyperemia^[10]^ (Figure 1). The aim of this study was to determine and compare the effects of Bretschneider’s histidine-tryptophan-ketoglutarate (HTK) solution and cold blood cardioplegia (CBC), which are frequently used cardioplegia techniques in cardiac surgery, on systemic endothelial functions.

## METHODS

This is a prospectively designed interventional study. The study was designed as a randomized and controlled trial. The approvals of both the Necmettin Erbakan University, Meram Faculty of Medicine, Clinical Research Ethics Committee (28.02.2018 - 2018/160) and the Republic of Turkey, Ministry of Health, Turkish Medicines and Medical Devices Agency (71146310-511.06-E.49682 - clinical trial number) were obtained. The study was financed by the University Scientific Research Projects Coordination. Additionally, informed written consent for voluntary participation were obtained from the patients involved in the study.

**Fig. 2:**
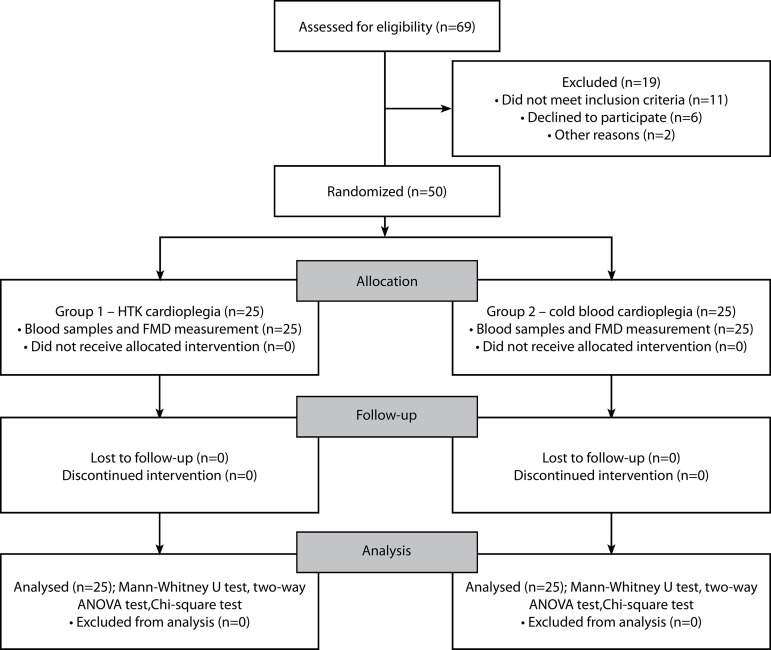
“Comparison between the effects of Bretschneider’s HTK solution and cold blood cardioplegia on systemic endothelial functions in patients who undergo coronary artery bypass surgery: A prospective randomized and controlled trial” – flow diagram. ANOVA=analysis of variance; FMD=flow-mediated dilation; HTK=histidine-tryptophan-ketoglutarate

A total of 50 patients with no gender preference that are between 40 and 80 years of age were included in the study conducted in Necmettin Erbakan University, Meram Faculty of Medicine, Department of Cardiovascular Surgery. The patients in whom an isolated coronary artery bypass grafting (CABG) procedure was scheduled to be performed under elective conditions were randomly divided using block randomization into two homogeneous groups consisting of 25 patients each. In patients of group 1 (n=25), Bretschneider’s HTK solution was administered, and in patients of group 2 (n=25), conventional CBC was administered. The presence of cardiac valve pathology, heart failure (left ventricular ejection fraction [LVEF] < 30%), chronic renal failure (creatine value > 2 mg/dl), impaired liver function test result, emergency surgery, redo surgery, positive history of cerebrovascular disease or carotid artery disease, and positive history of cardiopulmonary resuscitation were determined as the criteria for exclusion from the study. The study ended when 50 patients were reached (Figure 2).

**Fig. 3:**
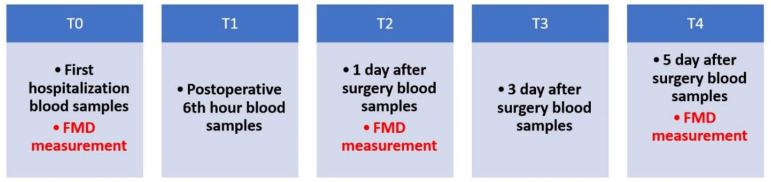
Diagram showing the time of taking samples and performing measurement of flow-mediated dilation (FMD) (%) values from patients included in the study.

In all patients, age, gender, body mass index (BMI), drug use, and the presence of comorbidities (diabetes mellitus, hypertension, hyperlipidemia, chronic obstructive pulmonary disease (COPD), asthma, peripheral arterial disease) were recorded preoperatively, together with biochemical parameters. Additionally, FMD values and blood values of ET-1, ADMA, vWF, and lactate were recorded. All data were recorded preoperatively (T0), and at day 0 (T1), day 1 (T2), day 3 (T3), and day 5 (T4) during the postoperative period (Figure 3).

Similar anesthetic technique was applied to all patients during the operation. All patients were operated by the same surgical team using the same surgical procedure. After the anesthesia protocol was completed, surgical procedure was started. Purse sutures were placed in the ascending aorta and right atrium appendix. The arterial cannula was placed proximal to the site of the innominate artery and venous cannulation was performed by two-stage cannula. The root cannula was placed in the aortic root and both venting and cardioplegia procedures were performed from this cannula. Antegrade cardioplegia was used in all cases. Mild to moderate systemic hypothermia was performed in all cases (30-34°C). When the appropriate temperature, activated clotting time (ACT) (Medtronic Inc., Minneapolis, Minnesota, USA) value, and flow were reached, a cross-clamp was placed and cardioplegia solution was given from the aortic root. Heating was started while internal mammary artery (IMA) - left anterior descending distal anastomosis was performed. Proximal anastomoses were performed to the appropriate area in the ascending aorta with side clamp.

Group 1 patients received Bretschneider’s HTK (Custodiol®, Germany) solution as a cardioplegia solution at a dose of 20 ml/kg. No additional dose was given until terminal warm cardioplegia was administered. Group 2 patients received conventional CBC at a dose of 15 ml/kg and were given maintenance cardioplegia every 20 minutes (10 ml/kg) after diastolic arrest. Before aortic declamping, normothermic blood “hot shot” was administered. One liter of CBC contains 22.2 mmol/L of potassium, 179.1 mmol/L of sodium, 18.6 mmol/L of magnesium, 2.8 mmol/L of calcium, a pH of 7.30-7.40, and a 4:1 blood:crystalloid ratio with cooling to 4 degrees. Both groups received terminal warm cardioplegia just before lifting the cross clamp.

Intraoperative data was recorded in all patients. After surgery, the patients were followed up in the intensive care unit (ICU) and transferred to the surgical ward as early as postoperative day 1. All patients were discharged from the hospital to full clinical recovery as early as postoperative day 5.

Blood samples were taken at times T0, T1, T2, T3, and T4 to determine endothelial dysfunction and sent to the cold chain biochemistry laboratory. Serums obtained by centrifugation of blood samples were stored until analyzed at -80°C. Human ET-1, human vWF, and ADMA levels were measured by the enzyme-linked immunosorbent assay (ELISA) technique. These kits use monoclonal antibodies against human ET-1, ADMA, and vWF (Elabscience Co., Houston, Texas, USA). Lactate levels were calculated using an amperometric metabolite sensor instrument (ABL700, Radiometer, Copenhagen, Denmark).

FMD measurement (Mindray M7-7.5 Mhz, United Kingdom), which is another indicator of endothelial function, was measured three times, as preoperative (T0), postoperative 1^st^ day (T2), and postoperative 5^th^ day (T4). FMD was measured from the dominant arm of the patient after six hours of fasting. Measurements were made considering the principles published by Münzel et al.^[10]^ (Figure 1).

### Statistical Analysis

Statistical analysis was performed using the IBM SPSS Statistics software (IBM® Inc., Chicago, USA), version 21.0. Descriptive statistics were summarized with frequency distributions and percentiles for categorical data and arithmetic mean ± standard deviation and median (minimum-maximum) for numerical data. The conformity of the variables with the normal distribution was investigated using visual (histogram and probability graphs) and analytical methods (Kolmogorov-Smirnov and Shapiro-Wilk tests). Numerical variables that were not having a normal distribution were compared using the Mann-Whitney U test and the two-way analysis of variance (ANOVA) for mixed measured. The Chi-square test was used to compare categorical data. A *P*-value <0.05 was considered statistically significant.

## RESULTS

The patients’ characteristics, demographic information, baseline data, and intraoperative data are listed in Table 1. The mean patient age was 61.4±8.6 years and the median age was 62 (39-77) years in both study groups. The female/male ratio of the patients involved in the study was 25%. The mean BMI was 28.7±4.0 and the median BMI was 28.1 (22.3-39.1). The mean LVEF was 53.9%±5.6 and the median LVEF was 55% (42-65%) in the whole patient group. When the two study groups were compared regarding the sociodemographic and medical data, no statistical differences were found in terms of age, gender, BMI, comorbidities, drugs used, and LVEF. In addition, ACT, aortic clamping time, duration of CPB and cross-clamp time, intraoperative urine volume, use of IMA, number of coronary lesions, and bypassed coronary arteries had similar results in both groups. The incidence of intraoperative whole blood use (fresh whole blood) was 4% in the Bretschneider’s HTK group and 8% in the CBC group. There was no difference between the groups in terms of intraoperative blood product use (*P*=0.600). When the aortic clamp was removed, spontaneous heartbeat occurred in 36% of the Bretschneider’s HTK group and fibrillated in 64% of these patients. In the CBC group, these rates were 68% and 32%, respectively. Spontaneous heartbeat was significantly higher in the CBC group (*P*=0.024).

**Table 1:** Patients' characteristics, demographic information, and baseline data.

Variables		Bretschneider's HTK	Cold blood cardioplegia	*P*-value
Age (mean ± SD)	(year)	60.1±7.8	62.7±9.4	0.183*
Sex, n (%)	Male	19 (76)	21 (84)	0.480**
	Female	6 (24)	4 (16)	
BMI (mean ± SD)	Kg/cm^2^	29.7±4.3	27.8±3.4	0.140*
LVEF (mean ± SD)	(%)	54.0±5.7	53.8±5.6	0.771*
Amount of cardioplegia (mean ± SD)*	(cc)	1285±196	1838±344	0.001*
Medical treatments	ACE inhibitor (+)	16 (64)	18 (72)	0.544**
	Beta-blocker (+)	23 (92)	25 (100)	0.149**
	Statin (+)	10 (40)	7 (28)	0.370**
	Diuretic (+)	7 (28)	6 (24)	0.747**
	Aspirin (+)	22 (88)	24 (96)	0.297**
Comorbid diseases	Type 2 DM (+)	12 (48)	13 (52)	0.777**
	HT (+)	17 (68)	12 (48)	0.152**
	Hyperlipidemia (+)	9 (36)	12 (48)	0.390**
	COPD (+)	3 (12)	3 (12)	1.000**
	PAD (+)	0	0	1.000**
Operative features	Dose of heparin (IU)	30.120±5.174	28.260±3.565	0.202*
	Dose of protamine (mg)	30.960±5.175	31.800±4.536	0.503*
	ACT (sec)	478±55	498±57	0.157*
	Pump output ACT value	114±6	112±12	0.259*
	Aortic clamp time (sec)	54.5±15.4	49.7±10.9	0.491*
	Cardiopulmonary bypass time (min)	94.2±23.7	93.3±21.1	0.719*
	Average heat CPB (ºC)	31.9±0.8	31.7±0.5	0.159*
	Side clamp time (min)	19.8±11.7	21.2±11.6	0.854*
	Heart re-beat type***			0,024**
	Spontaneously	9 (36)	17 (68)	
	Fibrillated	16 (64)	8 (32)	
	Cardiac arrest time (sec)	38.3±10.4	39.9±18.4	0.961*
	Intraoperative urine amount (cc)	385±242	339±126	0.876*
	Intraoperative blood product use (+)	1 (4)	2 (8)	0.600**
	Number of coronary lesions	3.0±0.7	3.1±0.7	0.734*
	Number of bypass	3.0±0.7	3.1±0.7	0.578*
	Use of IMA	25 (100)	25 (100)	1.000**

* Mann-Whitney U test

** Chi-square test

*** Heart re-beat type: after removal of the cross-clamp, the patients who needed defibrillation were identified as fibrillated and the group of patients who did not require defibrillation were identified as spontaneously.

ACE=angiotensin-converting enzyme; ACT=activated clotting time; BMI=body mass index; COPD=chronic obstructive pulmonary disease; CPB=cardiopulmonary bypass; DM=diabetes mellitus; HT=arterialhypertension; HTK=histidine-tryptophan-ketoglutarate; IMA=internal mammary artery; IU=international unit; LVEF=left ventricular ejection fraction; PAD=peripheral arterial disease; SD=standard deviation

ADMA, vWF, ET-1, FMD, and lactate levels were compared with two-way ANOVA for mixed measured for both groups. A significant difference was found between five different measurements of four indicators (*P*=0.001) and three measurements of one indicator (FMD) (*P*<0.001). ET-1 levels were significantly different between the groups (P=0.002). Indicator-group interaction was present for vWF and ET-1 levels (respectively, *P*=0.043 and *P*=0.001). Details of the analysis are presented in Table 2. Lactate levels were higher in HTK group than in CBC group. However, this difference was not statistically significant (*P*=0.301).

**Table 2:** Distribution of the levels of endothelial dysfunction indicators in Bretschneider's HTK and cold blood cardioplegia groups according to the time points together with the comparison of the two cardioplegia groups.

		Bretschneider's HTK	Cold blood cardioplegia	F statistic	*P*-value*		F statistic	*P*-value*		F statistic	*P*-value*
ADMA (µmol/L)	T0	100±26	100±52	18.833	0.001	Group	2.59	0.115	ADMA group interaction	1.886	014
	T1	148±34	157±44								
	T2	140±57	149±41								
	T3	128±46	155±51								
	T4	121±50	145±52								
vWF (U/L)	T0	4.0±0.6	4.2±0.8	66.287	0.001	Group	0.019	0.892	vWF group interaction	2.79	0.043
	T1	5.9±1.2	6.3±1.2								
	T2	5.6±1.2	5.5±1.3								
	T3	5.1±1.1	4.7±1.0								
	T4	4.7±0.7	4.6±0.7								
ET-1 (pg/mL)	T0	38.7±12.5	36.6±5.2	99.115	0.001	Group	10.923	0.002	ET-1 group interaction	8.957	0.001
	T1	59.0±13.1	68.4±6.2								
	T2	56.5±14.0	66.6±6.4								
	T3	49.4±15.0	63.5±7.8								
	T4	45.5±11.0	53.5±10.2								
Lactate (mmol/L)	T0	1.4±0.4	1.4±0.4	25.32	0.001	Group	1.67	0.202	Lactate group interaction	1.23	0.301
	T1	2.5±0.5	2.1±1.1								
	T2	1.9±0.6	1.8±0.6								
	T3	1.7±0.5	1.4±0.4								
	T4	1.5±0.4	1.5±0.5								
FMD (%)	T0	6.20±0,92	6.10±1.14	46.083	0.001	Group	4.326	0.043	FMD group interaction	6.58	0.008
	T2	5.57±0.79	4.68±1.02								
	T4	5.74±0.81	5.27±0.89								

* Two-way analysis of variance for mixed measured

ADMA=asymmetric dimethylarginine; ET-1=endothelin-1; FMD=flow-mediated dilation; HTK=histidine-tryptophan-ketoglutarate; vWF=von Willebrand factor

**Fig. 4:**
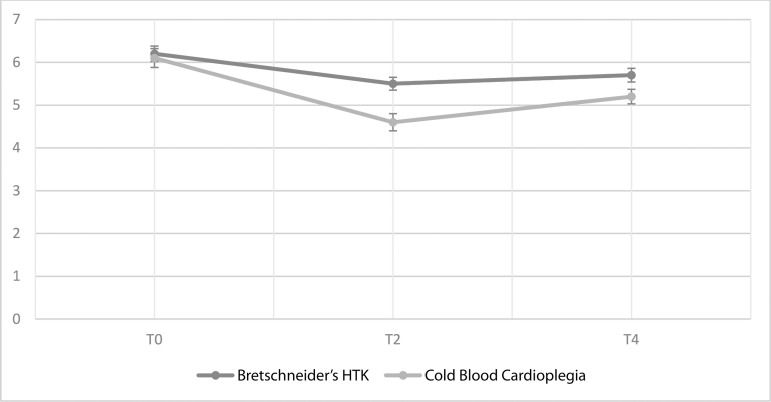
Distribution of the flow-mediated dilation levels of the study groups according to the time points (%). HTK = Histidine-tryptophan-ketoglutarate

Figure 4 shows the distribution of the levels of FMD in Bretschneider’s HTK and CBC groups according to the time points together with the comparison of the two cardioplegia groups. The FMD level was statistically significantly higher in the HTK group compared to the CBC group at time points T2 and T4 (*P*=0.002).

Table 3 shows the postoperative complications in HTK and CBC groups together with the comparison between them. There was no significant difference between Bretschneider’s HTK and CBC groups in terms of complications. Mortality was not observed in any of the 50 patients included in the study. In the HTK group, 28% had pulmonary complications (postoperative atelectasis in five patients, exacerbation of COPD in one patient, pulmonary edema in one patient), antibiotic revision was required in 12% (no sepsis was observed), temporary arrhythmia was observed in 8% (atrial fibrillation [AF] in two), neurologic complications in 8% (delirium in two), renal complications in 2% (temporary urea and creatinine elevation), and gastrointestinal complications in 4% (dyspepsia). In the CBC group, 28% had pulmonary complications (postoperative atelectasis in five patients, exacerbation of COPD in one patient, pulmonary edema in one patient), antibiotic revision was required in 32% (no sepsis was observed), temporary arrhythmia was observed in 20% (AF in five), neurologic complications in 8% (delirium in two), renal complications in 2% (temporary urea and creatinine elevation), and gastrointestinal complications in 4% (dyspepsia).

**Table 3:** Postoperative complications.

		Bretschneider's HTK	Cold blood cardioplegia	*P*-value
Total complications	n (%)	11 (44)	13 (52)	0.571
Pulmonary	n (%)	7 (28)	7 (28)	1.000
Antibiotic revision	n (%)	3 (12)	8 (32)	0.088
Arrhythmia	n (%)	2 (8)	5 (20)	0.221
Neurological	n (%)	2 (8)	2 (8)	1.000
Renal	n (%)	2 (8)	2 (8)	1.000
Gastrointestinal	n (%)	1 (4)	0	0.312

HTK=histidine-tryptophan-ketoglutarate

Table 4 includes patient follow-up information in the postoperative period. There was no significant difference between the two groups in terms of total drainage, postoperative blood product use (erythrocyte suspension), mean inotropic agent requirement, extubation time, and ICU stay.

**Table 4:** Patient follow-up in the postoperative period.

		Bretschneider's HTK	Cold blood cardioplegia	*P*-value
Amount of drainage	(cc)	1027±362	1123±450	0.600
Amount of blood used	unit	1.7±1.1	1.7±1.4	0.819
Inotropic need and dosage	mcg/kg/day	0.039±0.058	0.040±0.053	0.460
Extubation time	(hour)	9.6±2.7	10.8±4.2	0.335
ICU stay	(hour)	42.8±17.4	46.4±19.7	0.366

HTK=histidine-tryptophan-ketoglutarate; ICU=ıntensive care unit

## DISCUSSION

HTK is an intracellular cardioplegic solution that contains important antioxidants and buffers as well as low sodium concentration to prepare the heart by inhibiting the rapid phase of action potential^[13]^. It may be considered that functions of the components of HTK solution might play a role in the superiority of Bretschneider’s HTK solution in terms of endothelial injury. While histidine, which is present in the HTK solution, increases anaerobic glycolysis, tryptophan stabilizes the cell membrane, and mannitol decreases cellular edema. Histidine, a buffer with protein structure, has properties superior to bicarbonate in terms of balancing the intracellular pH and preservation of adenosine triphosphate store^[14,15]^.

The number of studies comparing Bretschneider’s HTK solution and CBC regarding endothelial injury is limited, and those were mostly studies conducted on animals. Our study prospectively evaluated endothelial injury caused by cardioplegia solutions in humans comprehensively for the first time. The most significant results of our study are that the level of ET-1, which is among the indicators of endothelial dysfunction, has a higher trend, and the FMD level is lower in the CBC group when compared to the HTK group. Our study reveals that Bretschneider’s HTK solution caused less endothelial injury than CBC. When we review recent clinical studies, we find that FMD is the most studied parameter with the highest prognostic value^[16]^. On the other hand, the absence of statistically significant differences between vWF and ADMA levels in related groups shows that the difference between the two cardioplegia solutions is not extreme in terms of endothelial injury.

In the study recently conducted by Li et al.^[17]^ (2017), the effects of HTK on pulmonary arterial perfusion and lung protection during CPB were investigated. Their patients were divided into two groups, the HTK group and the control group. In the HTK group, the pulmonary artery was perfused with HTK, and its effects on the levels of interleukin-6, malonaldehyde, and ET-1 were compared with the control group. It was reported that ET-1 level increased in both groups, but the ET-1 level was lower in the HTK group than in the control group. In a study conducted by Yang et al.^[18]^ (2004), HTK solution was compared with Wisconsin University (WU) solution in porcine hearts. In this study, endothelial functions were evaluated by using endothelium-derived hyperpolarizing factor (EDHF), which is used to improve the vascular tone of endothelium; EDHF-mediated endothelial functions of HTK solution were reported to be better than WU solution. In the study conducted by Saitoh et al. in rats^[19]^, HTK solution was compared to WU solution. When vascular structures were evaluated *in vitro* by dissecting coronary arteries, it was determined that HTK solution was more successful in protecting coronary vascular structures and preservation of the relaxation response to sodium nitroprusside after reperfusion.

In our study, when time-related intra-group changes regarding endothelial injury due to cardioplegia solutions were assessed, it was found that in both groups, the levels of endothelial injury indicators such as ADMA, vWF, ET-1, and lactate increased, and the brachial artery FMD value, which was expected to be reduced in endothelial injury, decreased following cardiac surgery. On the other hand, the levels of endothelial damage indicators decreased on postoperative 1^st^, 3^rd^, and 5^th^ days and FMD value increased as time progressed.

Our results suggested that cardiac surgery was related to endothelial injury, as confirmed by other previously conducted studies. In a 2017 study by Brettner F. et al.^[5]^, 30 patients who underwent elective coronary artery bypass surgery were assigned to the conventional coronary artery bypass group (n=15) and to the off-pump CABG group (n=15). They examined angiopoietin-1, angiopoietin-2, vascular endothelial-cadherin, and endocan at various time points to measure endothelial deterioration. They found significant increases in all measured parameters compared to the baseline values in both study groups. These results indicate endothelial deterioration and activation of endothelial cells in patients undergoing major cardiac surgery. The quantitative deviation of the parameters in conventional surgery compared to the off-pump surgery group shows that there is a relationship between ischemia/reperfusion formation and the degree of endothelial activation. Skrabal et al.^[2]^ evaluated endothelial damage in CABG operation^[2]^. In their study, they observed that circulating endothelial cells, vWF, and thrombomodulin levels did not change 30 minutes after the onset of CPB. However, they found out that the parameters began to increase and then began to decrease later in the postoperative period. Therefore, they suggested that endothelial damage may be associated with ischemia/reperfusion injury. The course of the parameters that we evaluated in our study pointing to endothelial dysfunction supports this study.

Another finding of our study was that the lactate level was higher in the HTK group than in the CBC group. However, this difference between lactate levels is not statistically significant. Hyperlactatemia is frequently seen after cardiac surgery. According to the studies, serum lactate level > 3 mmol/L is recommended as an indicator of the increased risk of morbidity and mortality^[20]^. In a prospective study of 1,820 patients published by Kogan A. et al.^[21]^, the blood lactate value was evaluated in predicting postoperative mortality, ventilation duration, and length of ICU stay (secondary outcome). According to the study, blood lactate levels > 4.4 mmol/L were found to be associated with prolonged ventilation time, longer ICU stay, and increased mortality^[21]^. However, the mean lactate value in the HTK group at time T1 was 2.5 ± 0.5 mmol/L. We think that there is no clinically significant reflection.

In our study, there was significantly more spontaneous ventricular fibrillation after release of cross-clamping in the HTK group. In a retrospective study published by Prathanee S. et al.^[22]^, CABG patients were divided into two groups, blood cardioplegia and HTK group, according to the type of cardioplegia used. Defibrillation after CABG was performed in 8.3% of the blood cardioplegia group and in 33.8% of the HTK group. Other postoperative complications were similar in both groups. The HTK group had significantly more spontaneous ventricular fibrillation after aortic declamping than the blood cardioplegia group. It has been suggested that the increase in the frequency of fibrillation is associated with low levels of adenosine triphosphate, differences in electrolyte concentrations between cell membranes, reperfusion, and oxidative stress damage^[23,24]^. When the postoperative complications were evaluated, the safety profile of both solutions was similar in our study.

### Limitations and Highlights

Our study had several limitations. First, the number of patients was relatively small. This small number of patients may be responsible for the lack of statistical significance between ADMA, vWF, and lactate levels between the groups. Second, even though more than one parameter among the endothelial injury indicators were evaluated, it is not possible to consider that all factors causing endothelial injury were evaluated. However, this problem was attempted to be overcome by the random selection of patients for cardioplegia groups. On the other hand, the subject of endothelial injury with HTK solution has not been investigated in detail in previously conducted clinical studies. Since our study is one of the limited studies in human on the effects of cardioplegia varieties on endothelial function, our results should be carefully interpreted and confirmed by other possible randomized trials in the future.

## CONCLUSION

Endothelial activation and dysfunction play an important role in morbidity and mortality after CPB as a reason for the development and continuation of systemic inflammation. ADMA, vWF, ET-1, and FMD were preferred for investigation of endothelial injury. While ADMA and vWF levels were found to be similar, the postoperative ET-1 level was determined to be higher, and the FMD value was lower in the CBC group. For this reason, it was found that the endothelial injury value was lower with HTK solution than with CBC. Therefore, our results suggest that less severe endothelial injury with Bretschneider’s HTK solution compared to CBC might be a factor for preferring HTK solution rather than CBC in CPB operations. However, when the complication rates were analyzed, it was observed that both cardioplegia solutions had a similar safety profile.

**Table:** 

Authors' roles & responsibilities	
IM YD CT OT MI NG EYO	Substantial contributions to the conception or design of the work; or the acquisition, analysis, or interpretation of data for the work; final approval of the version to be published Substantial contributions to the conception or design of the work; or the acquisition, analysis, or interpretation of data for the work; final approval of the version to be published Substantial contributions to the conception or design of the work; or the acquisition, analysis, or interpretation of data for the work; final approval of the version to be published Drafting the work or revising it critically for important intellectual content; final approval of the version to be published Final approval of the version to be published Agreement to be accountable for all aspects of the work in ensuring that questions related to the accuracy or integrity of any part of the work are appropriately investigated and resolved; final approval of the version to be published Agreement to be accountable for all aspects of the work in ensuring that questions related to the accuracy or integrity of any part of the work are appropriately investigated and resolved; final approval of the version to be published
